# Avifauna of the El Pambilar Wildlife Refuge in Ecuador

**DOI:** 10.3897/BDJ.13.e147753

**Published:** 2025-04-29

**Authors:** Edith Montalvo, Javier Pianchiche, Alex Garofalo, José Bonilla, Freddy Gallo-Viracocha, Luis D Montalvo

**Affiliations:** 1 Programa de Conservación de Cóndor Andino, Quito, Ecuador Programa de Conservación de Cóndor Andino Quito Ecuador; 2 Laboratorio de Ornitología, Departamento de Biología, Escuela Politécnica Nacional, Quito, Ecuador Laboratorio de Ornitología, Departamento de Biología, Escuela Politécnica Nacional Quito Ecuador; 3 Oficina Técnica, Refugio de Vida Silvestre el Pambilar, Ministerio del Ambiente, Agua y Transición Ecológica, Las Golondrinas, Ecuador Oficina Técnica, Refugio de Vida Silvestre el Pambilar, Ministerio del Ambiente, Agua y Transición Ecológica Las Golondrinas Ecuador; 4 Fundación Ecología Neotropical, Quito, Ecuador Fundación Ecología Neotropical Quito Ecuador; 5 Universidad de Florida, Gainesville, United States of America Universidad de Florida Gainesville United States of America

**Keywords:** El Pambilar Wildlife Refuge, tropical forest, avifauna, dataset

## Abstract

**Background:**

The Pambilar Wildlife Refuge (RVSP) is a well-preserved forest on the northern coast of Ecuador and an important refuge for birds, particularly for species with distributions restricted to the Chocó. Data were collected over 83 days from 7 October 2013 to 28 August 2019. This dataset contains 3,638 records, representing 116 orders, 39 families, 120 genera and 158 species.

**New information:**

This dataset represents the first article published on the avifauna of the El Pambilar Wildlife Refuge (RVSP) including information collected from 2013 to 2019. It includes taxonomic data collected over several years, as well as detailed information on the location, sampling effort and dates of the surveys. All data are available at the Global Biodiversity Information Facility (GBIF).

## Introduction

The Chocó-Darién ecoregion has been internationally recognised as one of the areas with the greatest biodiversity on the Planet. This ecoregion covers northwest South America and mainly includes the humid tropical and montane forests of eastern Panama, the Pacific slope of Colombia and northwest Ecuador (WWF Colombia 2008). In particular, in the Ecuadorian Chocó, there are approximately 10,000 species of vascular plants, which represents around 25% of the flora recorded in Ecuador. Between 13% and 20% of these species are endemic, highlighting the uniqueness and fragility of this ecosystem (Vázquez et al. 2005).

The Chocó Region is notable not only for its biodiversity, but also for its high endemism, with a total of 257 endemic species having been identified, including 162 plants and 95 species of fauna, amongst which are 57 species of amphibians, 18 of reptiles and 20 of birds (Bonilla and Valoyes 2020a, Bonilla and Valoyes 2020b).

Despite its immense biological and cultural importance, the Chocó-Darién ecoregional complex faces significant threats. Since the second half of the 20^th^ century, natural landscapes have undergone an accelerated transformation, mainly due to the conversion of forest into grasslands or land destined for crops (WWF Colombia 2008). These changes are driven by the expansion of the road network and economic integration processes, both nationally and transnationally, which have resulted in the loss and fragmentation of habitats. In Ecuador, the pressures from logging companies in search of new production areas are especially concerning.

The Ecuadorian Chocó, recognised globally for its biodiversity and high rates of endemism, faces serious threats, mainly due to deforestation caused by activities such as logging, agricultural expansion, land-use conversion and mining (Palacios and Jaramillo 2016, Conservation International 2019). In 1970, more than 80% of the region was covered by forest. However, in recent decades, the growing demand for wood products and land conversion into African palm and shrimp farms, have caused the loss of about 85% of the original forest cover (Sierra et al. 2003).

**Figure 1.**
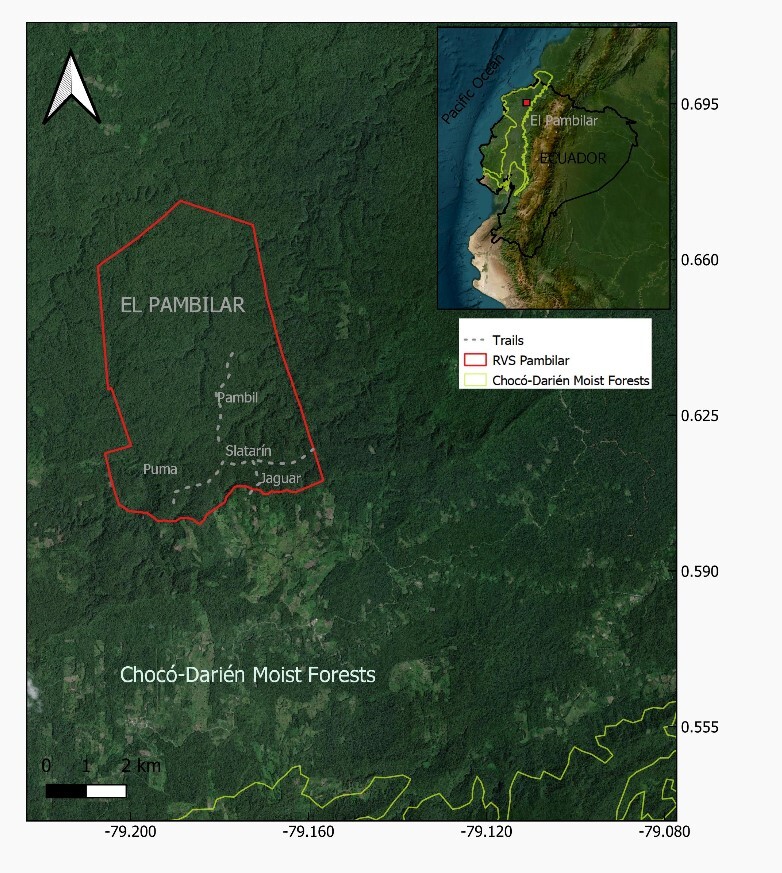
Location of El Pambilar Wildlife Refuge (RVS Pambilar) in Ecuador (top right, red star) and its boundaries (red polygon). The refuge is situated within the Chocó-Darién Moist Forests region (yellow polygon). The four trails surveyed within the RVS Pambilar are marked with dotted lines: Puma, Pambil, Saltarin, and Jaguar.

The RVSP is located in the Province of Esmeraldas, in northwest Ecuador (Fig. 1). It covers an area of 3,123 hectares and ranges between 200 and 360 m above sea level (Ministerio del Ambiente 2011). The RVSP aims to contribute to the conservation of the tropical humid forests in the Chocó Region. Notable for its high humidity and exceptional biodiversity, the RVSP stands out as a key area for conservation of biodiversity (Ministerio del Ambiente 2011).

Given the threats and exceptional diversity of the Chóco-Darién, it is important to implement conservation measures to protect these ecosystems and ensure the survival of their unique biodiversity.

## Sampling methods

### Sampling description

The data were obtained from observations carried out over 87 days by Javier Pianchiche (JP), Alex Garofalo (AG) and José Bonilla (JB), park rangers of the El Pambilar Wildlife Refuge (RVSP). These records were complemented by Edith Montalvo (EM), researcher at the National Polytechnic School and Luis Daniel Montalvo (LDM), researcher at the University of Florida.

Each census consisted of observations conducted along the trails in the early morning or late afternoon under favourable weather conditions. Each census lasted between two and three days and was repeated on subsequent dates. Observations were made using Zeiss 8 x 40 or Bushnell 8 x 30 binoculars. The Xeno-canto website was used for identifying and comparing bird songs.

Data collection was carried out using a combination of methods and observers, including mist netting, transect surveys and point counts. The RVSP features four trails: Pambil, Puma, Saltarín and Jaguar, each approximately 2 km in length. On the Jaguar trail, 10 mist nets (12 m x 3 m) were deployed for six days, while transects were established along the Pambil, Puma and Saltarín trails and surveyed over a period of 83 days.

The data were collected from 7 October 2013 to 28 August 2019, as detailed below:

Park Rangers JP, AG and JB conducted audiovisual recordings on the Puma, Pambil and Saltarín trails from 6:00 a.m. to 11:00 a.m. on the following dates:

- 2013, sampling occurred over 8 days. The observation dates were 7, 17 and 28 October; and 8, 18, 19 and 27 November as well as 6 December.
- 2017, observations took place over 21 days on the following dates: 20, 21, 26, 27, 28 and 29 September; 3, 4, 24 and 25 October; and 7, 8, 14, 15, 16 and 17 November ; as well as 12, 13, 14, 15 and 16 December.
- 2018, there were 27 sampling days on the following dates: 17, 29 and 30 May; 13, 14, 19 and 20 June; 12, 24, 25 and 30 July; 28 August; 1, 12, 13 and 21 September; and 1, 10, 16, 17, 24 and 25 October; additionally, on 10, 13, 14, 15 and 28 .
- 2019, observations occurred over 19 days on the following dates: 16, 17, 19, 29 and 30 January; 19 and 26 February; 26 and 27 March; 16 and 24 April; and 21, 22, 23, 25 and 26 May; as well as 10 and 17 July and 28 August.

Audiovisual recordings by LDM were conducted along the Jaguar and Pambilar trails between 6:00 a.m. and 1:00 p.m. on 28 and 29 July 2016, 2 and 3 August 2017 and 17 and 18 April 2018. Mist net captures performed by EM took place on 1, 2, 3 and 4 August 2017, as well as 17 and 18 April 2018, from 6:00 a.m. to 6:00 p.m.

A Canon EOS 90D DSLR camera was used to photograph birds and their habitats. The identification and classification of the species were carried out using the "Field Guides to Birds of Ecuador" by Ridgely and Greenfield (2001) and Freile and Restall (2018). The final dataset was organised in the Darwin Core format and uploaded to GBIF upon completion of data collection.

## Geographic coverage

### Description

The RVSP covers an area of 31.22 km² (Ministerio del Ambiente 2017). Data collection was carried out on one-third of the total area of RSVP, using trails ranging in length from 1.5 km on the Jaguar to 2.5 km on the Pambil Trail. The habitat corresponds to Evergreen Forest ecosystem of the Equatorial Chocó Lowlands (Ministerio del Ambiente 2012). Data were collected covering all habitats of the Reserve, including the Tropical Lowland Evergreen Forest, the Floodplain Tropical Evergreen Forest, the River Edge Forest and the Montane Forest Evergreen (Stotz et al. 1996).

### Coordinates

0.607312 and -79.174986 Latitude; -79.174951 and 0.640864 Longitude.

## Taxonomic coverage

### Description

The RVSP, one of the few well-preserved forest remnants found on the northern coast of Ecuador, is an important habitat for birds, especially for the species with distributions restricted to the Chocó. This dataset includes 3,638 records from 16 orders, 39 families, 120 genera and 158 species (Table 1). The nomenclature follows the South American Classification Committee (SACC) (Remsen et al. 2024).

According to Ridgely and Greenfield (2001), 19 endemic species occur in the Chocó ecoregion: *Crypturellusberlepschi* (Rothschild, 1897), *Penelopeortoni* Salvin, 1874, *Patagioenasgoodsoni* (Hartert, E, 1902), *Nyctiphrynusrosenbergi* (Hartert, 1895), *Phaethornisyaruqui* (Bourcier, 1851), *Amaziliarosenbergi* (Boucard, 1895), *Trogoncomptus* Zimmer, 1948, *Capitoquinticolor* Elliot, 1865, *Ramphastosbrevis* Meyer de Schauensee, 1945, *Piculuslitae* (Rothschild, 1901), *Micrasturplumbeus* Sclater, WL, 1918, *Pyriliapulchra* Berlepsch, 1897, *Myrmecizaberlepschi* (Hartert, 1898), *Pittasomarufopileatum* Hartert, 1901, *Rhynchocycluspacificus* (Chapman, 1914), *Cephalopteruspenduliger* Sclater, PL, 1859, *Tanagerjohannae* (Dalmas, 1900), *Dacnisberlepschi* Hartert, 1900 and *Chlorothraupisolivacea* (Cassin, 1860). According to these authors, two endemic species occur on the western Andean slope, including the high parts of Chocó: *Tangarapalmeri* (Hellmayr, 1909) and *Chrysothlypissalmoni* Sclater, PL, 1886. In addition, *Veniliorniscallonotus* (Waterhouse, 1841) and *Campephilusgayaquilensis* (Lesson, 1845) are considered endemic to the Tumbes Region.

The national threat categories were obtained from the Red List of Freile et al. (2019). In contrast, the global threat categories were obtained from IUCN (2024).

Amongst the 56 species in the RSVP included in the national Red List, nine are classified as Endangered (EN). Particularly, *Penelopeortoni* (Salvin, 1874) is listed as EN both nationally and globally, *Rhynchortyxcinctus* (Salvin, 1876) and *Capitoquinticolor* (Elliot, 1865) are considered EN at the national level, but are classified as Near Threatened (NT) globally. Similarly, *Micrasturplumbeus* (Sclater, 1918), *Cephalopteruspenduliger* (Sclater, 1859) and *Dacnisberlepschi* (Hartert, 1900) are considered as Endangered nationally, but classified as Vulnerable (VU) globally. Interestingly, *Amazonaautumnalis* (Linnaeus, 1758), *Phaenostictusmcleannani* (Lawrence, 1860) and *Pittasomarufopileatum* (Hartert, 1901) are only listed as Endangered in the national Red List.

Eleven species are considered Vulnerable (VU) at the local level. These include *Crypturellusberlepschi* (Rothschild, 1897), *Odontophoruserythrops* (Gould, 1859), *Trogonmassena* (Gould, 1838), *Campephilusgayaquilensis* (Lesson, 1845), *Pyriliapulchra* (Berlepsch, 1897), *Myrmecizaberlepschi* (Hartert, 1898), *Hylopezusperspicillatus* (Lawrence, 1861), *Sclerurusguatemalensis* (Hartlaub, 1844) and *Lipaugusunirufus* (Sclater, 1860). Furthermore, *Penelopepurpurascens* (Wagler, 1830) and *Cryptoleucopteryxplumbea* (Salvin, 1872) are classified as VU locally, but are considered Near Threatened (NT) globally.

Additionally, 36 species are listed as Near Threatened (NT) at the national level: *Tinamusmajor* (Gmelin, 1789); *Sarcoramphuspapa* (Linnaeus, 1758); *Patagioenasgoodsoni* (Hartert, E, 1902); *Megascopsguatemalae* Sharpe, 1875; *Nyctiphrynusrosenbergi* (Hartert, 1895); *Androdonaequatorialis* Gould, 1863; *Amaziliarosenbergi* (Boucard, 1895); *Trogoncomptus* Zimmer, 1948; *Trogoncaligatus* Gould, 1838; *Jacameropsaureus* (Statius Müller, 1776); *Ramphastosambiguus* Swainson, 1823; *Ramphastosbrevis* Meyer de Schauensee, 1945; *Pteroglossustorquatus* Gmelin, JF, 1788; *Piculuslitae* (Rothschild, 1901); *Campephilushaematogaster* (Tschudi, 1844); *Amazonafarinosa* (Boddaert, 1783); *Dysithamnuspuncticeps* Salvin, 1866; *Hafferiazeledoni* Ridgway, 1909; *Hylophylaxnaevioides* (Lafresnaye, 1847); *Formicariusnigricapillus* Ridgway, 1893; *Dendrocolaptessanctithomae* (Lafresnaye, 1852); *Xiphorhynchuslachrymosus* (Lawrence, 1862); *Xiphorhynchuserythropygius* (Sclater, PL, 1860); *Rhynchocycluspacificus* (Chapman, 1914); *Rhytipternaholerythra* (Sclater & Salvin, 1860); *Ceratopipramentalis* Sclater, PL, 1857; *Laniocerarufescens* (Sclater, PL, 1858); *Cantorchilusleucopogon* Salvadori & Festa, 1899; *Cyphorhinusphaeocephalus* Sclater, PL, 1860; *Turdusobsoletus* Lawrence, 1862; *Turdusassimilis* Cabanis, 1851; *Tangarapalmeri* (Hellmayr, 1909); *Tangarajohannae* (Dalmas, 1900); *Chrysothlypissalmoni* Sclater, PL, 1886; *Chlorothraupisolivacea* (Cassin, 1860) and *Spizaetusornatus* (Daudin, 1800) that is also classified as NT globally.

*Elanoidesforficatus* exhibits boreal migration as well as resident populations according to Ridgely and Greenfield (2001). No other migratory species were recorded.

### Taxa included

**Table:** 

Rank	Scientific Name	Common Name
kingdom	Animalia	Animals
class	Aves	Birds
order	Accipitriformes	
order	Apodiformes	
order	Caprimulgiformes	
order	Cathartiformes	
order	Columbiformes	
order	Coraciiformes	
order	Falconiformes	
order	Galbuliformes	
order	Galliformes	
order	Gruiformes	
order	Passeriformes	
order	Piciformes	
order	Psittaciformes	
order	Strigiformes	
order	Tinamiformes	
order	Trogoniformes	
family	Accipitridae	
family	Bucconidae	
family	Capitonidae	
family	Caprimulgidae	
family	Cardinalidae	
family	Cathartidae	
family	Columbidae	
family	Conopophagidae	
family	Cotingidae	
family	Cracidae	
family	Falconidae	
family	Formicariidae	
family	Fringillidae	
family	Furnariidae	
family	Galbulidae	
family	Grallariidae	
family	Hirundinidae	
family	Icteridae	
family	Incertae Sedis	
family	Momotidae	
family	Odontophoridae	
family	Parulidae	
family	Picidae	
family	Pipridae	
family	Polioptilidae	
family	Psittacidae	
family	Rallidae	
family	Ramphastidae	
family	Strigidae	
family	Thamnophilidae	
family	Thraupidae	
family	Tinamidae	
family	Tityridae	
family	Trochilidae	
family	Troglodytidae	
family	Trogonidae	
family	Turdidae	
family	Tyrannidae	
family	Vireonidae	
species	*Accipiterbicolor* (Vieillot, 1817)	
species	*Amaziliarosenbergi* (Boucard, 1895)	
species	*Amazonaautumnalis* (Linnaeus, 1758)	
species	*Amazonafarinosa* (Boddaert, 1783)	
species	*Androdonaequatorialis* Gould, 1863	
species	*Atticoratibialis* Cassin, 1853	
species	*Attilaspadiceus* (Gmelin, JF, 1789)	
species	*Automolusochrolaemus* (Tschudi, 1844)	
species	*Automolussubulatus* Spix, 1824	
species	*Baryphthengusmartii* (Spix, 1824)	
species	*Buteogallusurubitinga* (Gmelin, JF, 1788)	
species	*Cacicusuropygialis* Lafresnaye, 1843	
species	*Campephilusgayaquilensis* (Lesson, 1845)	
species	*Campephilushaematogaster* (Tschudi, 1844)	
species	*Campylorhamphustrochilirostris* (Lichtenstein, MHK, 1820)	
species	*Campylorhynchuszonatus* (Lesson, 1832)	
species	*Cantorchilusleucopogon* Salvadori & Festa, 1899	
species	*Cantorchilusnigricapillus* Sclater, PL, 1860	
species	*Capitoquinticolor* Elliot, 1865	
species	*Cephalopteruspenduliger* Sclater, PL, 1859	
species	*Ceratopipramentalis* Sclater,PL, 1857	
species	*Cercomacroidestyrannina* Sclater, PL, 1855	
species	*Chlorestesjulie* (Bourcier, 1842)	
species	*Chlorophanesspiza* (Linnaeus, 1758)	
species	*Chlorothraupisolivacea* (Cassin, 1860)	
species	*Chrysothlypissalmoni* Sclater, PL, 1886	
species	*Coerebaflaveola* (Linnaeus, 1758)	
species	*Cryptoleucopteryxplumbea* Salvin, 1872	
species	*Cryptopipoholochlora* Sclater,PL, 1888	
species	*Crypturellusberlepschi* (Rothschild, 1897)	
species	*Crypturellussoui* (Hermann, 1783)	
species	*Cyanerpescaeruleus* (Linnaeus, 1758)	
species	*Cyanerpescyaneus* (Linnaeus, 1766)	
species	*Cyphorhinusphaeocephalus* Sclater, PL, 1860	
species	*Dacnisberlepschi* Hartert, 1900	
species	*Dendrocinclafuliginosa* (Vieillot, 1818)	
species	*Dendrocolaptessanctithomae* (Lafresnaye, 1852)	
species	*Dryocopuslineatus* (Linnaeus, 1766)	
species	*Dysithamnuspuncticeps* Salvin, 1866	
species	*Elanoidesforficatus* (Linnaeus, 1758)	
species	*Electronplatyrhynchum* (Leadbeater, 1829)	
species	*Epinecrophyllafulviventris* Lawrence, 1862	
species	*Eubuccobourcierii* (Lafresnaye, 1845)	
species	*Euphoniasaturata* (Cabanis, 1860)	
species	*Euphoniaxanthogaster* Sundevall, 1834	
species	*Eutoxeresaquila* (Bourcier, 1847)	
species	*Formicariusnigricapillus* Ridgway, 1893	
species	*Geotrygonmontana* (Linnaeus, 1758)	
species	*Glyphorynchusspirurus* (Vieillot, 1819)	
species	*Grallariaguatimalensis* Prévost & Des Murs, 1842	
species	*Gymnopithysleucaspis* (Sclater, PL, 1855)	
species	*Hafferiazeledoni* Ridgway, 1909	
species	*Harpagusbidentatus* (Latham, 1790)	
species	*Hemithraupisguira* (Linnaeus, 1766)	
species	*Henicorhinaleucosticta* (Cabanis, 1847)	
species	*Herpetotherescachinnans* (Linnaeus, 1758)	
species	*Hylopezusperspicillatus* (Lawrence, 1861)	
species	*Hylophylaxnaevioides* (Lafresnaye, 1847)	
species	*Jacameropsaureus* (Statius Muller, 1776)	
species	*Laniocerarufescens* (Sclater, PL, 1858)	
species	*Laterallusalbigularis* (Lawrence, 1861)	
species	*Lepidothrixcoronata* Spix, 1825	
species	*Leptodoncayanensis* (Latham, 1790)	
species	*Leptopogonsuperciliaris* Tschudi, 1844	
species	*Lipaugusunirufus* Sclater, PL, 1860	
species	*Lophostrixcristata* (Daudin, 1800)	
species	*Lophotriccuspileatus* (Tschudi, 1844)	
species	*Malacoptilapanamensis* Lafresnaye, 1847	
species	*Manacusmanacus* (Linnaeus, 1766)	
species	*Megascopsguatemalae* Sharpe, 1875	
species	*Melanerpespucherani* (Malherbe, 1849)	
species	*Micrasturplumbeus* Sclater, WL, 1918	
species	*Micrasturruficollis* (Vieillot, 1817)	
species	*Micrastursemitorquatus* (Vieillot, 1817)	
species	*Microbatescinereiventris* (Sclater, PL, 1855)	
species	*Microcerculusmarginatus* (Sclater, PL, 1855)	
species	*Microrhopiasquixensis* (Cornalia, 1849)	
species	*Mionectesoleagineus* (Lichtenstein, 1823)	
species	*Mionectesolivaceus* Lawrence, 1868	
species	*Myiarchustuberculifer* (d'Orbigny & Lafresnaye, 1837)	
species	*Myiophobusfasciatus* (Statius Muller, 1776)	
species	*Myiornisatricapillus* (Lawrence, 1875)	
species	*Myiothlypisfulvicauda* Spix, 1825	
species	Myiozetetescayanensis (Linnaeus, 1766)	
species	*Myiozetetessimilis* (Spix, 1825)	
species	*Myrmecizaberlepschi* (Hartert, 1898)	
species	*Myrmecizaexsul* Sclater, PL, 1859	
species	*Myrmotherulaaxillaris* (Vieillot, 1817)	
species	*Myrmotherulaignota* Griscom, 1929	
species	*Myrmotherulapacifica* Hellmayr, 1911	
species	*Notharchushyperrhynchus* Sclater, PL, 1856	
species	*Nyctiphrynusrosenbergi* (Hartert, 1895)	
species	*Odontophoruserythrops* Gould, 1859	
species	*Ornithionbrunneicapillus* Lawrence, 1862	
species	*Pachysylviadecurtata* Bonaparte, 1838	
species	*Patagioenasgoodsoni* (Hartert, E, 1902)	
species	*Patagioenassubvinacea* Lawrence, 1868	
species	*Penelopeortoni* Salvin, 1874	
species	*Penelopepurpurascens* Wagler, 1830	
species	*Phaenostictusmcleannani* (Lawrence, 1860)	
species	*Phaethornisguy* (Lesson, 1833)	
species	*Phaethornisyaruqui* (Bourcier, 1851)	
species	*Pheugopediusmystacalis* Sclater, PL, 1860	
species	*Phyllomyiasgriseiceps* (Sclater, PL & Salvin, 1871)	
species	*Piculuslitae* (Rothschild, 1901)	
species	*Pionuschalcopterus* (Fraser, 1841)	
species	*Pionusmenstruus* (Linnaeus, 1766)	
species	*Pittasomarufopileatum* Hartert, 1901	
species	*Pteroglossustorquatus* Gmelin, JF, 1788	
species	*Pulsatrixperspicillata* (Latham, 1790)	
species	*Pyriliapulchra* Berlepsch, 1897	
species	*Pyrrhuramelanura* (Spix, 1824)	
species	*Querulapurpurata* (Statius Muller, 1776)	
species	*Ramphastosambiguus* Swainson, 1823	
species	*Ramphastosbrevis* Meyer de Schauensee, 1945	
species	*Ramphocelusflammigerus* Jardine & Selby, 1833	
species	*Rhynchocycluspacificus* (Chapman, 1914)	
species	*Rhynchortyxcinctus* (Salvin, 1876)	
species	*Rhytipternaholerythra* (Sclater & Salvin, 1860)	
species	*Saltatorgrossus* (Linnaeus, 1766)	
species	*Saltatormaximus* (Statius Müller, PL, 1776)	
species	*Sarcoramphuspapa* (Linnaeus, 1758)	
species	*Schiffornisveraepacis* (Sclater, PL; Salvin, O 1860)	
species	*Sclerurusguatemalensis* (Hartlaub, 1844)	
species	*Sclerurusobscurior* Sclater,PL, 1857	
species	*Spizaetusornatus* (Daudin, 1800)	
species	*Strixnigrolineata* Sclater,PL, 1859	
species	*Strixvirgata* Cassin, 1849	
species	*Tachyphonusdelatrii* Lafresnaye, 1847	
species	*Tachyphonusluctuosus* d'Orbigny & Lafresnaye, 1837	
species	*Tangaracyanicollis* (d'Orbigny & Lafresnaye, 1837)	
species	*Tangaragyrola* (Linnaeus, 1758)	
species	*Tangarajohannae* (Dalmas, 1900)	
species	*Tangaralarvata* (Du Bus De Gisignies, 1846)	
species	*Tangarapalmeri* (Hellmayr, 1909)	
species	*Thaluraniacolombica* Bourcier, 1843	
species	*Thraupisepiscopus* (Linnaeus, 1766)	
species	*Thraupispalmarum* (Wied, 1821)	
species	*Threnetesruckeri* (Bourcier, 1847)	
species	*Tinamusmajor* (Gmelin, 1789)	
species	*Tityracayana* (Linnaeus, 1766)	
species	*Troglodytesaedon* Vieillot, 1809	
species	*Trogoncaligatus* Gould, 1838	
species	*Trogonchionurus* Sclater, PL & Salvin, 1871	
species	*Trogoncollaris* Vieillot, 1817	
species	*Trogoncomptus* Zimmer, 1948	
species	*Trogonmassena* Gould, 1838	
species	*Trogonrufus* Gmelin, 1788	
species	*Turdusassimilis* Cabanis, 1851	
species	*Turdusobsoletus* Lawrence, 1862	
species	*Tyrannuluselatus* (Latham, 1790)	
species	*Tyrannusmelancholicus* Vieillot, 1819	
species	*Veniliorniscallonotus* (Waterhouse, 1841)	
species	*Veniliorniskirkii* (Malherbe, 1845)	
species	*Vireolaniusleucotis* (Swainson, 1837)	
species	*Xiphorhynchuserythropygius* (Sclater, PL, 1860)	
species	*Xiphorhynchuslachrymosus* (Lawrence, 1862)	
species	*Zimmeriuschrysops* (Sclater, PL, 1859)	

## Usage licence

### Usage licence

Creative Commons Public Domain Waiver (CC-Zero)

## Data resources

### Data package title

Avifauna del Refugio de vida silvestre El Pambilar

### Resource link

https://doi.org/10.60545/87dmuq

### Alternative identifiers

https://www.gbif.org/es/dataset/7482c011-1692-412d-a87c-220a579ccfd2

### Number of data sets

1

### Data set 1.

#### Data set name

Avifauna del Refugio de vida silvestre El Pambilar

#### Data format

Darwin core Archive

#### Download URL

https://www.gbif.org/es/dataset/7482c011-1692-412d-a87c-220a579ccfd2

#### Data format version

2.1

#### Description

Our occurrence data include 28 columns and all records are georeferenced (Montalvo et al. 2024).

**Data set 1.** 

Column label	Column description
id	Name of the trail where the data were taken.
type	The nature of the presence record, taxon or event.
language	A language of the resource.
licence	A legal document giving official permission to do something with the resource.
basisOfRecord	The specific nature of the data record.
occurrenceID	An identifier for the Occurrence. Record in the dataset or collection.
behaviour	The behaviour shown by the organism at the time of being registered.
eventID	An identifier for the set of information associated with an event (something that occurs at a place and time). It can be a globally unique identifier or a dataset-specific identifier.
eventTime	The time or interval during which the Event occurred. It is recommended to use an encoding scheme.
year	The four-digit year in which the Event occurred, according to the Common Era Calendar.
month	The integer month in which the Event occurred.
day	The integer day of the month on which the Event occurred.
verbatimEventDate	The date or interval during which an Event occurred.
country	The name of the country or major administrative unit in which the Location occurs.
countryCode	The standard code for the country in which the Location occurs.
stateProvince	The name of the next smaller administrative region than country (state, province, canton, department, region etc.) in which the Location occurs.
locality	The specific description of the place.
decimalLatitud	The geographic latitude (in decimal degrees, using the spatial reference system given in geodeticDatum) of the geographic centre of a Location. Positive values are north of the Equator, negative values are south of it. Legal values lie between -90 and 90, inclusive.
decimalLongitud	The geographic longitude (in decimal degrees, using the spatial reference system given in geodeticDatum) of the geographic centre of a Location. Positive values are east of the Greenwich Meridian, negative values are west of it. Legal values lie between -180 and 180, inclusive.
taxonID	An identifier for the set of taxon information (data associated with the Taxon class). May be a global unique identifier or an identifier specific to the dataset.
scientificName	An identifier for the nomenclatural (not taxonomic) details of a scientific name.
kingdom	The full scientific name of the kingdom in which the taxon is classified.
phylum	The full scientific name of the phylum or division in which the taxon is classified.
class	The full scientific name of the class in which the taxon is classified.
order	The full scientific name of the order in which the taxon is classified.
family	The full scientific name of the family in which the taxon is classified.
genus	The full scientific name of the genus in which the taxon is classified.
specificEpithet	The name of the first or species epithet of the scientificName.

## Figures and Tables

**Table 1.** Bird List for El Pambilar Wildlife Refuge.

Rank	Order	Family	Scientific name	The number of individual birds observed
1	Tinamiformes	Tinamidae	*Tinamusmajor* (Gmelin, 1789)	64
2	Tinamiformes	Tinamidae	*Crypturellusberlepschi* (Rothschild, 1897)	21
3	Tinamiformes	Tinamidae	*Crypturellussoui* (Hermann, 1783)	2
4	Galliformes	Cracidae	*Penelopeortoni* Salvin, 1874	2
5	Galliformes	Cracidae	*Penelopepurpurascens* Wagler, 1830	32
6	Galliformes	Odontophoridae	*Odontophoruserythrops* Gould, 1859	27
7	Galliformes	Odontophoridae	*Rhynchortyxcinctus* (Salvin, 1876)	22
8	Cathartiformes	Cathartidae	*Sarcoramphuspapa* (Linnaeus, 1758)	1
9	Accipitriformes	Accipitridae	*Leptodoncayanensis* (Latham, 1790)	1
10	Accipitriformes	Accipitridae	*Elanoidesforficatus* (Linnaeus, 1758)	3
11	Accipitriformes	Accipitridae	*Spizaetusornatus* (Daudin, 1800)	2
12	Accipitriformes	Accipitridae	*Harpagusbidentatus* (Latham, 1790)	1
13	Accipitriformes	Accipitridae	*Accipiterbicolor* (Vieillot, 1817)	3
14	Accipitriformes	Accipitridae	*Cryptoleucopteryxplumbea* Salvin, 1872	14
15	Accipitriformes	Accipitridae	*Buteogallusurubitinga* (Gmelin, JF, 1788)	3
16	Gruiformes	Rallidae	*Laterallusalbigularis* (Lawrence, 1861)	4
17	Columbiformes	Columbidae	*Patagioenassubvinacea* Lawrence, 1868	57
18	Columbiformes	Columbidae	*Patagioenasgoodsoni* (Hartert, E, 1902)	101
19	Columbiformes	Columbidae	*Geotrygonmontana* (Linnaeus, 1758)	1
20	Strigiformes	Strigidae	*Megascopsguatemalae* Sharpe, 1875	2
21	Strigiformes	Strigidae	*Lophostrixcristata* (Daudin, 1800)	1
22	Strigiformes	Strigidae	*Pulsatrixperspicillata* (Latham, 1790)	3
23	Strigiformes	Strigidae	*Strixvirgata* Cassin, 1849	3
24	Strigiformes	Strigidae	*Strixnigrolineata* Sclater,PL, 1859	1
25	Caprimulgiformes	Caprimulgidae	*Nyctiphrynusrosenbergi* (Hartert, 1895)	2
26	Apodiformes	Trochilidae	*Eutoxeresaquila* (Bourcier, 1847)	3
27	Apodiformes	Trochilidae	*Threnetesruckeri* (Bourcier, 1847)	3
28	Apodiformes	Trochilidae	*Phaethornisyaruqui* (Bourcier, 1851)	32
29	Apodiformes	Trochilidae	*Phaethornisguy* (Lesson, 1833)	1
30	Apodiformes	Trochilidae	*Androdonaequatorialis* Gould, 1863	6
31	Apodiformes	Trochilidae	*Thaluraniacolombica* Bourcier, 1843	3
32	Apodiformes	Trochilidae	*Amaziliarosenbergi* (Boucard, 1895)	3
33	Apodiformes	Trochilidae	*Chlorestesjulie* (Bourcier, 1842)	3
34	Trogoniformes	Trogonidae	Trogonmassena Gould, 1838	1
35	Trogoniformes	Trogonidae	*Trogoncomptus* Zimmer, 1948	4
36	Trogoniformes	Trogonidae	*Trogonchionurus* Sclater, PL & Salvin, 1871	35
37	Trogoniformes	Trogonidae	*Trogoncaligatus* Gould, 1838	1
38	Trogoniformes	Trogonidae	*Trogonrufus* Gmelin, 1788	4
39	Trogoniformes	Trogonidae	*Trogoncollaris* Vieillot, 1817	10
40	Coraciiformes	Momotidae	*Electronplatyrhynchum* (Leadbeater, 1829)	7
41	Coraciiformes	Momotidae	*Baryphthengusmartii* (Spix, 1824)	89
42	Galbuliformes	Galbulidae	*Jacameropsaureus* (Statius Muller, 1776)	10
43	Galbuliformes	Bucconidae	*Notharchushyperrhynchus* Sclater, PL, 1856	1
44	Galbuliformes	Bucconidae	*Malacoptilapanamensis* Lafresnaye, 1847	47
45	Piciformes	Capitonidae	*Capitoquinticolor* Elliot, 1865	1
46	Piciformes	Capitonidae	*Eubuccobourcierii* (Lafresnaye, 1845)	1
47	Piciformes	Ramphastidae	*Ramphastosambiguus* Swainson, 1823	131
48	Piciformes	Ramphastidae	*Ramphastosbrevis* Meyer de Schauensee, 1945	74
49	Piciformes	Ramphastidae	*Pteroglossustorquatus* Gmelin, JF, 1788	21
50	Piciformes	Picidae	*Melanerpespucherani* (Malherbe, 1849)	7
51	Piciformes	Picidae	*Veniliorniskirkii* (Malherbe, 1845)	4
52	Piciformes	Picidae	*Veniliorniscallonotus* (Waterhouse, 1841)	6
53	Piciformes	Picidae	*Piculuslitae* (Rothschild, 1901)	1
54	Piciformes	Picidae	*Dryocopuslineatus* (Linnaeus, 1766)	8
55	Piciformes	Picidae	*Campephilushaematogaster* (Tschudi, 1844)	2
56	Piciformes	Picidae	*Campephilusgayaquilensis* (Lesson, 1845)	32
57	Falconiformes	Falconidae	*Herpetotherescachinnans* (Linnaeus, 1758)	8
58	Falconiformes	Falconidae	*Micrasturruficollis* (Vieillot, 1817)	3
59	Falconiformes	Falconidae	*Micrasturplumbeus* Sclater, WL, 1918	2
60	Falconiformes	Falconidae	*Micrastursemitorquatus* (Vieillot, 1817)	5
61	Psittaciformes	Psittacidae	*Pyriliapulchra* Berlepsch, 1897	43
62	Psittaciformes	Psittacidae	*Pionusmenstruus* (Linnaeus, 1766)	37
63	Psittaciformes	Psittacidae	*Pionuschalcopterus* (Fraser, 1841)	6
64	Psittaciformes	Psittacidae	*Amazonaautumnalis* (Linnaeus, 1758)	14
65	Psittaciformes	Psittacidae	*Amazonafarinosa* (Boddaert, 1783)	233
66	Psittaciformes	Psittacidae	*Pyrrhuramelanura* (Spix, 1824)	1
67	Passeriformes	Thamnophilidae	*Dysithamnuspuncticeps* Salvin, 1866	20
68	Passeriformes	Thamnophilidae	*Epinecrophyllafulviventris* Lawrence, 1862	3
69	Passeriformes	Thamnophilidae	*Myrmotherulaignota* Griscom, 1929	5
70	Passeriformes	Thamnophilidae	*Myrmotherulapacifica* Hellmayr, 1911	1
71	Passeriformes	Thamnophilidae	*Myrmotherulaaxillaris* (Vieillot, 1817)	83
72	Passeriformes	Thamnophilidae	*Microrhopiasquixensis* (Cornalia, 1849)	1
73	Passeriformes	Thamnophilidae	*Cercomacroidestyrannina* Sclater, PL, 1855	3
74	Passeriformes	Thamnophilidae	*Myrmecizaexsul* Sclater, PL, 1859	185
75	Passeriformes	Thamnophilidae	*Myrmecizaberlepschi* (Hartert, 1898)	11
76	Passeriformes	Thamnophilidae	*Hafferiazeledoni* Ridgway, 1909	3
77	Passeriformes	Thamnophilidae	*Gymnopithysleucaspis* (Sclater, PL, 1855)	47
78	Passeriformes	Thamnophilidae	*Hylophylaxnaevioides* (Lafresnaye, 1847)	60
79	Passeriformes	Thamnophilidae	*Phaenostictusmcleannani* (Lawrence, 1860)	20
80	Passeriformes	Conopophagidae	*Pittasomarufopileatum* Hartert, 1901	9
81	Passeriformes	Grallariidae	*Grallariaguatimalensis* Prévost & Des Murs, 1842	2
82	Passeriformes	Grallariidae	*Hylopezusperspicillatus* (Lawrence, 1861)	34
83	Passeriformes	Formicariidae	*Formicariusnigricapillus* Ridgway, 1893	48
84	Passeriformes	Furnariidae	*Sclerurusobscurior* Sclater,PL, 1857	11
85	Passeriformes	Furnariidae	*Sclerurusguatemalensis* (Hartlaub, 1844)	3
86	Passeriformes	Furnariidae	*Dendrocinclafuliginosa* (Vieillot, 1818)	52
87	Passeriformes	Furnariidae	*Glyphorynchusspirurus* (Vieillot, 1819)	43
88	Passeriformes	Furnariidae	*Dendrocolaptessanctithomae* (Lafresnaye, 1852)	1
89	Passeriformes	Furnariidae	*Xiphorhynchuslachrymosus* (Lawrence, 1862)	31
90	Passeriformes	Furnariidae	*Xiphorhynchuserythropygius* (Sclater, PL, 1860)	1
91	Passeriformes	Furnariidae	*Campylorhamphustrochilirostris* (Lichtenstein, MHK, 1820)	1
92	Passeriformes	Furnariidae	*Automolusochrolaemus* (Tschudi, 1844)	3
93	Passeriformes	Furnariidae	*Automolussubulatus* Spix, 1824	26
94	Passeriformes	Tyrannidae	*Phyllomyiasgriseiceps* (Sclater, PL & Salvin, 1871)	1
95	Passeriformes	Tyrannidae	*Tyrannuluselatus* (Latham, 1790)	1
96	Passeriformes	Tyrannidae	*Ornithionbrunneicapillus* Lawrence, 1862	2
97	Passeriformes	Tyrannidae	*Zimmeriuschrysops* (Sclater, PL, 1859)	1
98	Passeriformes	Tyrannidae	*Mionectesolivaceus* Lawrence, 1868	87
99	Passeriformes	Tyrannidae	*Mionectesoleagineus* (Lichtenstein, 1823)	2
100	Passeriformes	Tyrannidae	*Leptopogonsuperciliaris* Tschudi, 1844	1
101	Passeriformes	Tyrannidae	*Myiornisatricapillus* (Lawrence, 1875)	1
102	Passeriformes	Tyrannidae	*Lophotriccuspileatus* (Tschudi, 1844)	1
103	Passeriformes	Tyrannidae	*Rhynchocycluspacificus* (Chapman, 1914)	1
104	Passeriformes	Tyrannidae	*Myiophobusfasciatus* (Statius Muller, 1776)	1
105	Passeriformes	Tyrannidae	*Myiozetetescayanensis* (Linnaeus, 1766)	1
106	Passeriformes	Tyrannidae	*Myiozetetessimilis* (Spix, 1825)	1
107	Passeriformes	Tyrannidae	*Tyrannusmelancholicus* Vieillot, 1819	1
108	Passeriformes	Tyrannidae	*Rhytipternaholerythra* (Sclater & Salvin, 1860)	28
109	Passeriformes	Tyrannidae	*Myiarchustuberculifer* (d'Orbigny & Lafresnaye, 1837)	1
110	Passeriformes	Tyrannidae	*Attilaspadiceus* (Gmelin, JF, 1789)	15
111	Passeriformes	Cotingidae	*Querulapurpurata* (Statius Muller, 1776)	24
112	Passeriformes	Cotingidae	*Cephalopteruspenduliger* Sclater, PL, 1859	112
113	Passeriformes	Cotingidae	*Lipaugusunirufus* Sclater, PL, 1860	42
114	Passeriformes	Pipridae	*Cryptopipoholochlora* Sclater,PL, 1888	8
115	Passeriformes	Pipridae	*Lepidothrixcoronata* Spix, 1825	159
116	Passeriformes	Pipridae	*Manacusmanacus* (Linnaeus, 1766)	13
117	Passeriformes	Pipridae	*Ceratopipramentalis* Sclater,PL, 1857	141
118	Passeriformes	Tityridae	*Tityracayana* (Linnaeus, 1766)	1
119	Passeriformes	Tityridae	*Schiffornisveraepacis* (Sclater, PL; Salvin, O 1860)	17
120	Passeriformes	Tityridae	*Laniocerarufescens* (Sclater, PL, 1858)	7
121	Passeriformes	Vireonidae	*Vireolaniusleucotis* (Swainson, 1837)	16
122	Passeriformes	Vireonidae	*Pachysylviadecurtata* Bonaparte, 1838	20
123	Passeriformes	Hirundinidae	*Atticoratibialis* Cassin, 1853	1
124	Passeriformes	Troglodytidae	*Microcerculusmarginatus* (Sclater, PL, 1855)	90
125	Passeriformes	Troglodytidae	*Troglodytesaedon* Vieillot, 1809	1
126	Passeriformes	Troglodytidae	*Campylorhynchuszonatus* (Lesson, 1832)	2
127	Passeriformes	Troglodytidae	*Pheugopediusmystacalis* Sclater, PL, 1860	15
128	Passeriformes	Troglodytidae	*Cantorchilusleucopogon* Salvadori & Festa, 1899	8
129	Passeriformes	Troglodytidae	*Cantorchilusnigricapillus* Sclater, PL, 1860	22
130	Passeriformes	Troglodytidae	*Henicorhinaleucosticta* (Cabanis, 1847)	14
131	Passeriformes	Troglodytidae	*Cyphorhinusphaeocephalus* Sclater, PL, 1860	13
132	Passeriformes	Polioptilidae	*Microbatescinereiventris* (Sclater, PL, 1855)	80
133	Passeriformes	Turdidae	*Turdusobsoletus* Lawrence, 1862	3
134	Passeriformes	Turdidae	*Turdusassimilis* Cabanis, 1851	157
135	Passeriformes	Thraupidae	*Tachyphonusluctuosus* d'Orbigny & Lafresnaye, 1837	2
136	Passeriformes	Thraupidae	*Tachyphonusdelatrii* Lafresnaye, 1847	237
137	Passeriformes	Thraupidae	*Ramphocelusflammigerus* Jardine & Selby, 1833	4
138	Passeriformes	Thraupidae	*Thraupisepiscopus* (Linnaeus, 1766)	6
139	Passeriformes	Thraupidae	*Thraupispalmarum* (Wied, 1821)	14
140	Passeriformes	Thraupidae	*Tangarapalmeri* (Hellmayr, 1909)	11
141	Passeriformes	Thraupidae	*Tangaralarvata* (Du Bus De Gisignies, 1846)	4
142	Passeriformes	Thraupidae	*Tangaracyanicollis* (d'Orbigny & Lafresnaye, 1837)	1
143	Passeriformes	Thraupidae	*Tangaragyrola* (Linnaeus, 1758)	1
144	Passeriformes	Thraupidae	*Tangarajohannae* (Dalmas, 1900)	2
145	Passeriformes	Thraupidae	*Dacnisberlepschi* Hartert, 1900	2
146	Passeriformes	Thraupidae	*Cyanerpescaeruleus* (Linnaeus, 1758)	2
147	Passeriformes	Thraupidae	*Cyanerpescyaneus* (Linnaeus, 1766)	1
148	Passeriformes	Thraupidae	*Chlorophanesspiza* (Linnaeus, 1758)	2
149	Passeriformes	Thraupidae	*Hemithraupisguira* (Linnaeus, 1766)	3
150	Passeriformes	Thraupidae	*Chrysothlypissalmoni* Sclater, PL, 1886	3
151	Passeriformes	Thraupidae	*Coerebaflaveola* (Linnaeus, 1758)	16
152	Passeriformes	Incertae Sedis	*Saltatormaximus* (Statius Müller, PL, 1776)	8
153	Passeriformes	Incertae Sedis	*Saltatorgrossus* (Linnaeus, 1766)	36
154	Passeriformes	Cardinalidae	*Chlorothraupisolivacea* (Cassin, 1860)	73
155	Passeriformes	Parulidae	*Myiothlypisfulvicauda* Spix, 1825	9
156	Passeriformes	Icteridae	*Cacicusuropygialis* Lafresnaye, 1843	16
157	Passeriformes	Fringillidae	*Euphoniasaturata* (Cabanis, 1860)	56
158	Passeriformes	Fringillidae	*Euphoniaxanthogaster* Sundevall, 1834	34
